# Effects of Dominant and Nondominant Limb Immobilization on Muscle Activation and Physical Demand during Ambulation with Axillary Crutches

**DOI:** 10.3390/jfmk6010016

**Published:** 2021-02-09

**Authors:** Kara B. Bellenfant, Gracie L. Robbins, Rebecca R. Rogers, Thomas J. Kopec, Christopher G. Ballmann

**Affiliations:** Department of Kinesiology, Samford University, Birmingham, AL 35229, USA; kbellenf@samford.edu (K.B.B.); grobbins@samford.edu (G.L.R.); rrogers1@samford.edu (R.R.R.); tkopec@samford.edu (T.J.K.)

**Keywords:** assistive device, electromyography, limb dominance, injury

## Abstract

The purpose of this study was to investigate the effects of how limb dominance and joint immobilization alter markers of physical demand and muscle activation during ambulation with axillary crutches. In a crossover, counterbalanced study design, physically active females completed ambulation trials with three conditions: (1) bipedal walking (BW), (2) axillary crutch ambulation with their dominant limb (DOM), and (3) axillary crutch ambulation with their nondominant limb (NDOM). During the axillary crutch ambulation conditions, the non-weight-bearing knee joint was immobilized at a 30-degree flexion angle with a postoperative knee stabilizer. For each trial/condition, participants ambulated at 0.6, 0.8, and 1.0 mph for five minutes at each speed. Heart rate (HR) and rate of perceived exertion (RPE) were monitored throughout. Surface electromyography (sEMG) was used to record muscle activation of the medial gastrocnemius (MG), soleus (SOL), and tibialis anterior (TA) unilaterally on the weight-bearing limb. Biceps brachii (BB) and triceps brachii (TB) sEMG were measured bilaterally. sEMG signals for each immobilization condition were normalized to corresponding values for BW.HR (*p* < 0.001) and RPE (*p* < 0.001) were significantly higher for both the DOM and NDOM conditions compared to BW but no differences existed between the DOM and NDOM conditions (*p* > 0.05). No differences in lower limb muscle activation were noted for any muscles between the DOM and NDOM conditions (*p* > 0.05). Regardless of condition, BB activation ipsilateral to the ambulating limb was significantly lower during 0.6 mph (*p* = 0.005) and 0.8 mph (*p* = 0.016) compared to the same speeds for BB on the contralateral side. Contralateral TB activation was significantly higher during 0.6 mph compared to 0.8 mph (*p* = 0.009) and 1.0 mph (*p* = 0.029) irrespective of condition. In conclusion, limb dominance appears to not alter lower limb muscle activation and walking intensity while using axillary crutches. However, upper limb muscle activation was asymmetrical during axillary crutch use and largely dependent on speed. These results suggest that functional asymmetry may exist in upper limbs but not lower limbs during assistive device supported ambulation.

## 1. Introduction

Lower extremity injuries have been shown to result in ≈14–15% of all annual emergency department visits in the United States (US) [1]. Ankle sprain/strains, lower limb contusions, and fractures are among the most common and often necessitate short-term use of assistive devices (AD) to aid ambulation [2]. Furthermore, chronic conditions and disabilities result in ≈6.1 million people in the US using some form of AD including crutches, canes, or walkers [3]. While necessary to maintain mobility either acutely or chronically, the use of ADs imposes higher metabolic demand and cardiorespiratory stress during ambulation versus able-bodied bipedal walking (BW) [4]. A multitude of evidence has substantiated findings of asymmetrical muscle activation during able-bodied BW, which may lead to inter-limb variability of metabolic demands [5,6]. However, it is currently unknown if functional limb asymmetry exists with the use of ADs during ambulation, which may have important implications for individuals recovering from an injury, undergoing rehabilitation, or with a chronic condition.

Functional limb asymmetry describes the theory whereby there are functional discrepancies between limbs during task completion [7]. Through this, it has been suggested that dominant and nondominant limbs serve differing roles during movement. During able-bodied BW, the dominant leg operates as the primary means for propulsion while the nondominant leg serves as support and stabilization [8]. While functional limb asymmetry has been supported by multiple groups, other findings have refuted or tempered the significance it has during locomotion. Polk et al. reported that braking and vertical propulsive forces are symmetrical between lower limbs, but that dominant limb ground reactive force (GRF) in the mediolateral directions are higher during ambulation than nondominant [6]. Supporting this, Gregg et al. showed that anteroposterior GRF of dominant limbs occurs at preferred walking speeds [9]. While the exact mechanisms for reports of asymmetry are not fully understood, previous evidence has suggested that differences in limb strength, muscle mass, and neuromuscular activation may have inter-limb variability [10,11]. However, the existence and practical importance of functional limb asymmetry is widely debated. Seeley et al. showed that, during slow and preferred walking speeds, dominant and nondominant limb propulsive impulses were relatively unchanged [7]. Propulsion of the dominant limb was greater at faster speeds, indicating a possibility for limb asymmetry at higher speeds but the functional consequences of this are not fully clear. No differences in symmetry index have been reported by others including gait parameters and sit-to-stand measurements in able-bodied individuals [12]. However, it should be noted that limb asymmetry has been suggested to be more prominent in individuals with disabilities or chronic conditions, further suggesting the need for research on limb asymmetry while using ADs and individuals with altered mobility [13,14].

The use of ADs for ambulation may be used acutely or chronically to promote functional mobility and can improve health outcomes and quality of life [3,15]. Several investigations have reported higher metabolic and cardiovascular demand associated with AD support during ambulation when compared to BW [4,16,17]. Annesley et al. showed increased heart rate and oxygen consumption while using rocker bottom and standard axillary crutches compared to BW [17]. In addition to axillary crutches, the use of standard and wheeled walkers have been shown to elicit a higher metabolic cost of ambulation compared to BW while the use of a single-point cane does not elicit significant increases in energy expenditure [4]. Collectively, these findings suggest that increased activation of upper body skeletal muscle and joint loading during assisted ambulation may contribute to the elicitation of increased physiological strain [18]. Reports of increases in psychophysiological factors including rate of perceived exertion (RPE) and attentional focus have also been shown to be altered during assisted ambulation [4,19]. However, the majority of evidence on AD ambulation either uses bilateral leg movement or assumes symmetry between limbs during ambulation. Given the widespread debate of functional limb symmetry during able-bodied BW, more investigation is needed to clarify the possible discrepancies in limb activation during ambulation with ADs.

While it is well supported that ambulation with axillary crutches increases physical and cardiovascular demand [14,16], nearly all investigations are standardized for the use of a single limb whether dominant or nondominant. Furthermore, many other investigations allow for participants to self-select speed during locomotion, thus being unable to standardize for pace. There has been considerable evidence suggesting limb asymmetry during able-bodied ambulation with inter-limb variability in force production and muscle activation [20,21]. Furthermore, others have suggested that this variability may be most evident in the lower leg joints and musculature [21,22]. Whether there are differences in muscle activation between limbs while using ADs with an immobilized limb is unknown. The purpose of this study was to elucidate how limb dominance and unilateral joint immobilization alter physical demands and muscle activation during ambulation with axillary crutches.

## 2. Materials and Methods

### 2.1. Study Design

**Figure 1:**
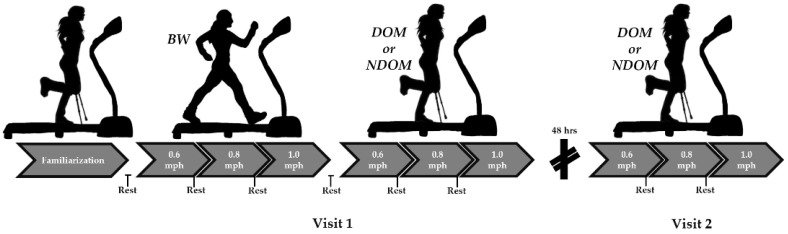
Study design. For visit 1, participants were familiarized with the axillary crutches followed by bipedal walking (BW). In a counterbalanced manner, participants then completed the dominant (DOM) or nondominant (NDOM) ambulation condition. Following a 48-h separation period, participants completed the 2nd visit, where they completed the final DOM or NDOM condition. For each treadmill bout, participants ambulated at 0.6, 0.8, and 1.0 mph phases. Each speed phase lasted 5 min. For all rest periods, participants sat until basal HR was reached before proceeding to the next phase. HR was monitored every minute, while sEMG was measured during the first 30 s and last 30 s of the speed phase.

An illustration of the study design can be seen in Figure 1. In a crossover counterbalanced study design, participants completed three ambulation conditions over two visits: (1) bipedal walking (BW), (2) axillary crutch-supported dominant limb ambulation with contralateral limb immobilization (DOM), and (3) axillary crutch-supported nondominant limb ambulation with contralateral limb immobilization (NDOM). Participants always completed the BW condition first, and the DOM and NDOM conditions were counterbalanced between visits. For each condition, participants ambulated at increased standardized speeds on a wide-belt treadmill: 0.6 mph, 0.8 mph, and 1.0 mph. For each speed, participants ambulated for 5 min and rested until their basal heart rate was attained prior to commencement of the next speed. Heart rate and rate of perceived exertion (RPE) were monitored every minute and averaged over the 5 min for analysis. Furthermore, surface electromyograph (sEMG) readings were recorded during the first and last 30 s of each speed to measure muscle activation of upper and weight bearing lower limbs. Visits were separated by a minimum of 48 h.

### 2.2. Participants

A convenience sample of 12 physically active female participants (age = 20.9 yrs ± 1.1, height = 163.5 cm ± 4.3, and body mass = 57.3 kg ± 6.2) were recruited for this investigation. Physically active was defined as participating in 150 min/week of moderate intensity exercise [23]. Safety of exercise was determined using a physical activity readiness questionnaire (PAR-Q). To be eligible to participate, all participants had to be free from an upper or lower body injury in the past six months, metabolic disease, cardiovascular disease, musculoskeletal disease, or other health problems. Additionally, participants were excluded from the study if they reported the use of an AD within the past 6 months. All subjects gave written and informed consent for inclusion before participation; the study was conducted after approval from the Samford University Institutional Review Board (IRB).

Prior to each exercise session, participants were asked to refrain from caffeine, nicotine, and alcohol 12 h prior and vigorous upper body exercise 24 h prior [23]. Prior to any data collection, verbal and written informed consent was obtained from each participant. All experimental procedures were conducted in accordance with the Declaration of Helsinki and approved by the Samford University Institutional Review Board (IRB) (EXPD-HP-20-SUM-16; 10 July 2020).

### 2.3. Surface Electromyograph (sEMG) Sensor Placement

**Figure 2:**
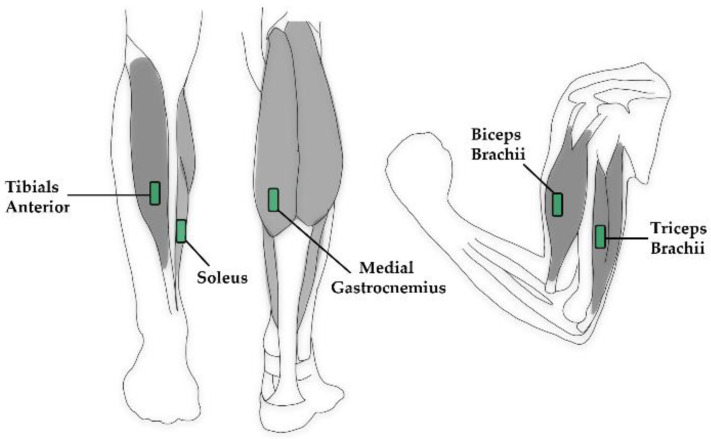
Illustration of surface electromyograph (sEMG) sensor (green) placement. For the lower limbs, the sensors were placed on the weight-bearing leg on the tibialis anterior, soleus, and medial gastrocnemius. For upper body limbs, the sensors were bilaterally placed on the biceps brachii and triceps brachii. All sensors were placed according to SENIAM recommendations [24].

To detect muscle activity during ambulation, wired sEMG sensors (SX230) connected to a Bluetooth EMG System (PS900) (Biometrics Ltd., Newport, UK) were used. During the BW condition, sensors on the lower limbs were placed bilaterally. For the DOM and NDOM conditions, sensors were placed unilaterally on the weight-bearing limb during ambulation. For all conditions, sensors were placed bilaterally on the upper limbs. Sensor placement on the lower and upper limbs is depicted in Figure 2. Plastic adhesive strips were attached to each sensor and pressed firmly onto the skin. Athletic pre-wrap was used to ensure good skin connection during activity and to reduce signal noise. All electrodes were placed according to surface EMG for a noninvasive assessment of muscles (SENIAM) recommendations [24]. The specific muscles and landmarks included the following:

▪ *Medial gastrocnemius* (MG): Participants laid prone with their knee fully extended; the sensor(s) were placed on the most prominent medial bulge of the muscle.
▪ *Soleus* (SOL): Participants sat upright wither their knee at 90 degrees in passive flexion with their heel on the floor; the sensors were placed at two thirds of the line between the medial condyle of the femur to the medial malleolus.
▪ *Tibialis anterior* (TA): Participants sat upright with their knee at 90 degrees in passive flexion with their heel on the floor; the sensors were placed at about a third on the line between the tip of the fibula and the tip of the medial malleolus.
▪ *Biceps brachii* (BB): Participants sat in a chair with the elbow passively flexed at 90 degrees and the dorsal side of the forearm in a horizontal position; the sensors were placed on the line between the medial acromion and the fossa cubit at one third from the fossa cubit.
▪ *Triceps brachii* (TB): Participants sat upright with their shoulder at approximately 90 degrees abduction with the arm 90 degrees flexed and the palm of the hand pointing downwards; the electrodes were placed half-way on the line between the posterior crista of the acromion and the olecranon at two finger widths medial to the line.
▪ *Ground strap*: A ground reference strap was placed on each medial malleolus.

### 2.4. Procedures

During the first session, each participant’s height and weight were recorded and lower limb dominance was obtained by the “kick a ball” test [25]. Briefly, a ball was placed on the ground and participants kicked the ball following the command of “kick this ball”, and whichever foot was used was deemed the dominant limb. Participants were then fitted with a HR monitor, and basal HR was documented (Polar Electro, Lake Success, NY, USA). Following this, aluminum axillary crutches (Mckesson, Irving, TX, USA) were fitted in the standing position according to the manufacturer’s recommendations; the height was adjusted to 5 cm below the participant’s axilla and was recorded to be used in the subsequent trial. The hand grips were adjusted to 25 degrees of flexion while shoulders were relaxed with the hands on the hand grips [26]. Participants were familiarized with using the axillary crutches and were asked to ambulate 10–20 m on the DOM and NDOM limbs. Form was corrected as needed, and participant verbal communication of comfortability using the crutches was confirmed.

Once fitted with all necessary monitoring equipment, participants completed 3 speed stages and walked at 0.6, 0.8, and 1.0 mph for 5 min each on a wide-belt motorized treadmill (Woodway, Waukesha, WI, USA). In between each speed stage, participants rested until basal HR was reached. During the BW condition, participants walked unassisted with both legs free. During the DOM and NDOM conditions, the respective dominant and nondominant legs were weight-bearing while the contralateral limb was immobilized using a hinged postoperative knee immobilizer brace (Breg, Carlsbad, CA, USA). The brace was attached proximally and distally to the knee joint at a fixed angle of 30 degrees to where the leg could not be used to bear weight. During ambulation, sEMG signals were recorded during the first and last 30 s of each speed stage. For analysis, the root mean square of the electrical signal (mV) of the DOM and NDOM conditions over the 30 s was standardized to corresponding speed stages for BW. Specifically, for upper limb activation analysis, muscle activation was measured bilaterally. Thus, the muscles were separated in relation to the side of the weight-bearing limb (i.e., contralateral bicep brachii activation for the DOM condition indicates the activation of the bicep brachii on the opposite side of the DOM leg used for weight bearing). Rate of perceived exertion (RPE; 6–20 Borg scale) was recorded at the ended of each speed phase. HR was documented every minute during ambulation and averaged over the entire 5-min stage.

### 2.5. Data Analysis

All data were analyzed using Jamovi software (Version 0.9). A 3 × 3 (condition × speed) repeated-measures ANOVA was used to detect statistical differences in markers of physical demand. For lower limb muscle activation, a 2 × 3 (condition × speed) repeated-measures ANOVA was used for analysis. A 2 × 2 × 3 (condition × muscle side × speed) repeated-measures ANOVA was used for upper limb muscle activation analysis. A Tukey’s post hoc analysis was used for multiple comparisons. Estimates of effect size for the main effects were calculated using eta squared (η^2^). Cohen’s d effect sizes were used for multiple comparisons and interpreted as 0.2—small, 0.5—moderate, and 0.8—large [27,28]. All data are presented as mean ± standard deviation (SD). Significance was set a *p* ≤ 0.05.

## 3. Results

### 3.1. Physical Demand Analysis

**Figure 3:**
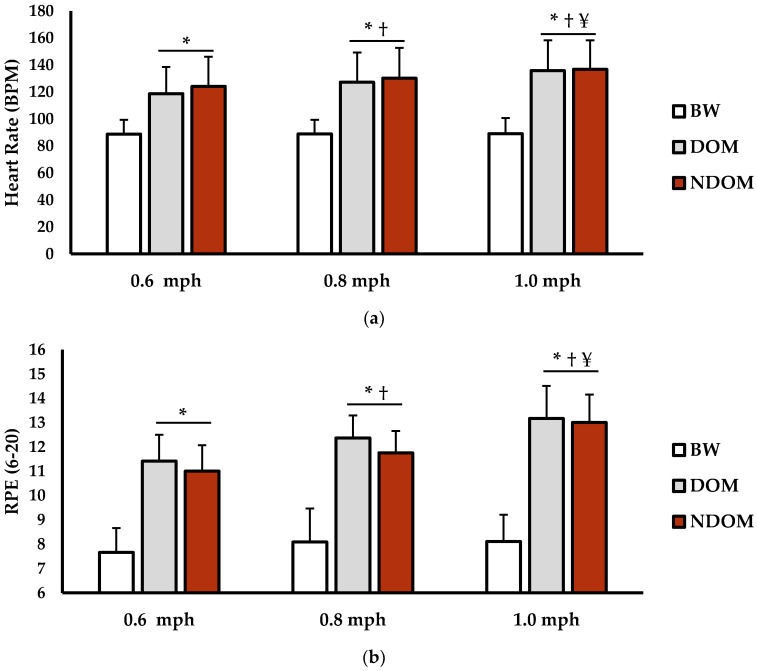
(**a**) Heart rate (bpm) for the 0.6, 0.8, and 1.0 mph speed phases between the bipedal walking (BW; white bars), dominant (DOM; light grey bars), and nondominant (NDOM; red bars) conditions. (**b**) Rate of perceived exertion (RPE; 6–20 scale) for the 0.6, 0.8, and 1.0 mph speed phases between the bipedal walking (BW; white bars), dominant (DOM; light grey bars), and nondominant (NDOM; red bars) conditions. The data are presented as mean ± SD. * indicates significantly different from BW (*p* < 0.05). † indicates significantly different from 0.6 mph (*p* < 0.05). ¥ indicates significantly different from 0.8 mph (*p* < 0.05).

Heart rate (HR) and rate of perceived exertion (RPE) can be seen in Figure 3. For HR (bpm), there was a main effect for speed (*p* < 0.001; η^2^ = 0.034), condition (*p* < 0.001; η^2^ = 0.611), and interaction for speed × condition (*p* < 0.001; η^2^ = 0.019). HR during 0.6 mph BW was significantly lower than during 0.6 mph DOM (*p* = 0.002; d = 3.62) and 0.6 mph NDOM (*p* < 0.001; d = 23.24). Additionally, HR during 0.8 mph BW was significantly lower than during 0.8 mph DOM (*p* < 0.001; d = 3.33) and 0.8 mph NDOM (*p* < 0.001; d = 3.72). HR during 1.0 mph BW was significantly lower during than 1.0 mph DOM (*p* < 0.001; d = 4.15) and 1.0 mph NDOM (*p* < 0.001; d = 4.37). For the DOM condition, HR was significantly lower during the 0.6 mph versus 0.8 mph (*p* < 0.001; d = 0.94) and 1.0 mph (*p* < 0.001; d = 1.45) speed phases. In the NDOM condition, HR was significantly lower during the 0.6 mph versus 0.8 mph (*p* < 0.001; d = 0.76) and 1.0 mph (*p* < 0.001; d = 1.80) speed phases. HR for 0.8 mph DOM was significantly lower versus 1.0 mph DOM (*p* < 0.001; d = 0.71). Lastly, HR for 0.8 NDOM was significantly lower than 1.0 NDOM (*p* < 0.001; d = 1.22). There were no significant differences for HR between DOM and NDOM for all speeds (*p* = 0.8629–0.999; d = 0.01–0.19).

For RPE (6–20 scale), there was a main effect for speed (*p* < 0.001; η^2^ = 0.057), condition (*p* < 0.001; η^2^ = 0.698), and interaction for speed × condition (*p* < 0.001; η^2^ = 0.010). RPE during 0.6 mph BW was significantly lower than during 0.6 mph DOM (*p* < 0.001; d = 1.95) and 0.6 mph NDOM (*p* < 0.001; d = 2.17). Furthermore, RPE during 0.8 mph BW was significantly lower than 0.8 mph DOM (*p* < 0.001; d = 2.36) and 0.8 mph NDOM (*p* < 0.001; d = 2.51). RPE during 1.0 mph BW was significantly lower than 1.0 mph DOM (*p* < 0.001; d = 2.74) and 1.0 mph NDOM (*p* < 0.001; d = 2.89). For the DOM condition, RPE was significantly lower during the 0.6 mph versus 0.8 mph (*p* < 0.001; d = 0.41) and 1.0 mph (*p* < 0.001; d = 0.80) speed phases. This was also seen in the NDOM condition, where RPE was significantly lower during the 0.6 mph versus 0.8 mph (*p* < 0.001; d = 0.28) and 1.0 mph (*p* < 0.001; d = 0.59) speed phases. RPE for 0.8 mph DOM was significantly lower versus 1.0 mph DOM (*p* = 0.049; d = 0.38). Lastly, RPE for 0.8 NDOM was significantly lower than 1.0 NDOM (*p* < 0.001; d = 0.30). There were no significant differences for RPE between DOM and NDOM for all speeds (*p* = 0.890–0.999; d = 0.01–0.12).

### 3.2. Muscle Activation Analysis

Muscle activation for lower limbs is presented in Table 1. For medial gastrocnemius activity (mV), there were no main effects for speed (*p* = 0.462; η^2^ = 0.014) or condition (*p* = 0.618; η^2^ = 0.008). Also, there was no interaction for speed × condition (*p* = 0.253; η^2^ = 0.048). Tibialis anterior activity (mV) showed no main effects speed (*p* = 0.544; η^2^ = 0.05) or condition (*p* = 0.806; η^2^ = 0.003). Additionally, there was no interaction for speed × condition (*p* = 0.714; η^2^ = 0.003). For soleus activity (mV), there were no main effects for speed (*p* = 0.713; η^2^ = 0.001) or condition (*p* = 0.947; η^2^ < 0.001). Also, there was no interaction for speed × condition (*p* = 0.307; η^2^ = 0.004).

Muscle activation for upper limb muscle is presented in Table 2. For biceps brachii activation (mV), there were main effects for speed (*p* = 0.022; η^2^ = 0.050) and muscle side (*p* < 0.001; η^2^ = 0.215) but not for condition (*p* = 0.856; η^2^ < 0.001). There were no significant interactions for condition × speed (*p* = 0.124; η^2^ = 0.006), speed × muscle side (*p* = 0.164; η^2^ = 0.050), or condition × muscle side (*p* = 0.085; η^2^ = 0.009). However, there was a significant interaction for condition × muscle side × speed (*p* = 0.019; η^2^ = 0.013). Multiple comparisons revealed that, for both DOM and NDOM conditions, ipsilateral biceps brachii (IBB) muscle activation was significantly lower during 0.6 mph (*p* = 0.005; d = 1.01) and 0.8 mph (*p* = 0.016; d = 0.79) compared to the corresponding speeds for the contralateral bicep brachii (CBB). No differences between IBB and CBB were found for 1.0 mph (*p* = 0.611; d = 0.11) Furthermore, IBB activation was significantly lower for 0.6 mph (*p* = 0.045; d = 0.65) and 0.8 mph (*p* = 0.048; d = 0.69) compared to 1.0 mph for both the DOM and NDOM conditions. No differences between 0.6 mph and 0.8 mph (*p* = 0.999; d < 0.01) were found for IBB. No differences existed between speeds for CBB activation (*p* = 0.758–0.999; d = 0.01–0.09).

For triceps brachii activation (mV), there was a main effect for speed (*p* < 0.001; η^2^ = 0.158) but not for condition (*p* = 0.208; η^2^ = 0.004) or muscle side (*p* = 0.456; η^2^ = 0.010). No significant interactions were found for condition × speed (*p* = 0.091; η^2^ = 0.005), speed × muscle side (*p* = 0.504; η^2^ = 0.001), condition × muscle side (*p* = 0.211; η^2^ = 0.004), or condition × muscle side × speed (*p* = 0.184; η^2^ = 0.003). Multiple comparisons revealed that, for the contralateral triceps brachii (CTB), activation during both the DOM and NDOM conditions was significantly higher during 0.6 mph versus 0.8 mph (*p* = 0.009; d = 1.24) and 1.0 mph (*p* = 0.029; d = 1.05). There were no differences between 0.8 mph and 1.0 mph (*p* = 0.998; d = 0.) for CTB activation. There were no differences between 0.6 mph and 0.8 mph (*p* = 0.131; d = 0.81) and 1.0 mph (*p* = 0.635; d= 0.51) for ipsilateral triceps brachii (ITB) activation for either condition. Additionally, no differences in ITB activation existed between 0.8 mph and 1.0 mph (*p* = 0.914; d = 0.47).

## 4. Discussion

The use of ADs for ambulation is common and has been shown to increase both cardiovascular and metabolic strain compared to BW [4,26,29]. While functional limb asymmetry between dominant and nondominant limbs has been documented during unassisted BW [6,12], no investigations to date have studied whether limb asymmetry exists with AD use and unilateral limb immobilization. Thus, the purpose of this study was to investigate how limb dominance and unilateral immobilization alter physical demands and muscle activation during ambulation with axillary crutches. These findings reveal that the use of axillary crutches with unilateral lower limb immobilization results in increased HR and RPE when compared to BW at various speeds. However, limb dominance had little effect on physical demand markers. Lower limb muscle activation during crutch-assisted ambulation was similar between speeds in the DOM and NDOM conditions. For upper limb muscle activation, IBB had lower activation compared to CBB and had lower activation at slower speeds independent of limb dominance. Furthermore, CTB had significantly lower activation at faster speeds regardless of limb dominance compared to ITB. These findings suggest that limb dominance does not differentially determine physical demand and lower limb muscle activation during axillary crutch use with unilateral limb immobilization. However, changes in upper limb muscle activation are different based on muscle orientation in relation to the weight-bearing limb during axillary crutch ambulation. While the precise mechanisms responsible for these changes are not fully clear from current data alone, these findings may have important implications for those recovering from a lower body injury or who have chronic conditions necessitating the use of ADs.

Increases in physical demand while using ADs have been reported by multiple groups, which supports the current findings [4,30]. Using crutches activates greater amounts of upper body muscle mass and has been shown to increase energy cost of the swing phase of gait versus BW [30]. This is further bolstered by other investigations showing that ADs that require greater muscle activation for support (i.e., standard walker) result in higher HRs than those that require lower muscle recruitment (i.e., single point cane) [4]. Indeed, Bhambani et al. showed that axillary crutch walking resulted in significantly higher HRs and RPE values compared to BW [31]. Since the use of axillary crutches potentiates the use of elbow flexors, shoulder flexors, and shoulder abductors [32], increases in HR and RPE are likely due to increased recruitment of the upper musculature compared to BW. However, the present data showed no differences in HR or RPE between the DOM and NDOM conditions. Previous evidence has shown that dominant and nondominant leg exercises at low loads result in similar ventilatory and cardiovascular responses when limbs are exercised independently [33]. The physiological mechanisms for similar physical demands between dominant and nondominant limbs in the current investigation are unclear. However, similar physical responses may be due to similar blood flow responses to the physical task. Evidence has shown a similar popliteal artery flow-mediated dilation during distal muscle activation between dominant and nondominant legs, suggesting comparable amounts of blood flow between limbs [34]. Furthermore, Jungmann et al. reported similar lower limb microvascular perfusion during BW indicative of increased bilateral leg muscle activation [35]. Although speculative, similar physical demand responses between limbs, namely HR, may be due to similar blood flow delivery. Since participants were working at similar workloads (i.e., speeds) while using the axillary crutches, the metabolic demand of the musculature in dominant and nondominant legs may have been similar, resulting in comparable amounts of blood flow. Neither blood flow nor muscle metabolism were measured, currently leaving the contribution of these to current findings unknown. However, previous evidence has linked lower limb perfusion and concomitant muscle activation both during walking and running, which supports this idea [35]. Future research is needed to determine if blood flow is different between dominant and nondominant limbs while using ADs and may provide further insight into how limb dominance influences muscle activation during assisted ambulation.

Muscle activation of the lower limbs was not different between the DOM and NDOM conditions while using axillary crutches. This is different from previous findings showing varied lower limb function during BW [6,8,9]. Disparities may be manifested in unilateral limb activation during axillary crutch ambulation versus bilateral activation during BW. During BW, motor neurons from the lumbar and sacral regions send neural signals to dominant and nondominant limbs differentially. Previous reports have suggested that neural activation and drive may be greater to the dominant limb during BW, thus partially explaining asymmetrical differences between limbs during BW [22]. During the DOM and NDOM conditions, only a single limb was used, thus not allowing for bilateral deficits during movement. Supporting the notion of differences in contractile function from unilateral to bilateral limb movements, previous reports have shown that maximal force exerted by a single leg during bilateral contractions were greater than unilateral contraction of the same leg [36]. Thus, it is plausible that recruitment of the same muscle may be different whether bilateral or unilateral contraction occurs, which could explain inconsistencies between BW and the present findings. A lack of lower limb differences may also be due to the health status of subjects. Participants in the present study were physically active, young, and healthy. Previous evidence has suggested that functional limb differences may be larger in individuals with chronic conditions or acute trauma [37]. For example, Dai et al. reported that patients showed severe kinematic asymmetries between injured legs 6 months after anterior cruciate ligament (ACL) reconstructive surgery both with and without protective knee bracing [37]. However, 48% of patients in their investigation injured their DOM limb and 52% injured their NDOM. This, as authors suggest, may show that limb asymmetries following trauma are more dependent on injury rather than limb dominance. However, it should be noted that the primary aim was to measure function of the injured limb, not the healthy supportive limb. Furthermore, it remains unclear if asymmetry with injury translates to limb function deficits while using axillary crutches or an AD.

Although there were no differences in lower limb activation, upper limbs were recruited differently depending on the speed of ambulation and which side/orientation the muscle was in relation to the weight-bearing limb. The IBB had less activation at slower speeds and, at these speeds, were activated less than the CBB. Past investigations have shown that elbow flexion and shoulder flexion increase simultaneously during crutched ambulation, particularly during the swing phase [32]. Current data suggest that, at lower speeds, increases in IBB activation (i.e., elbow flexion), which likely occurred during the swing phase, may not be necessary. However, the limb asymmetry of biceps brachii activation is less clear. Since the lower limb on the contralateral side was immobilized, this may have led to a greater need for isometric contralateral bicep contraction for support during movement on this side while ipsilateral bicep activity was able to decrease during slower speeds. In addition to this, the CTB also showed increased activity during slower speeds, suggesting simultaneous recruitment of agonist and antagonist muscles on the contralateral side. Isometric contractions are partially responsible for maintaining posture and balance while standing and during movement. Previous reports have shown that unilateral limb impairment in stroke patients causes weight-bearing asymmetries between limbs [38]. Related to crutch usage, Silva et al. reported that pressure loading while using crutches was significantly higher for the right versus left hand, albeit no limb immobilization was used [39]. Thus, it appears that, regardless of limb dominance, the use of axillary crutches with limb immobilization results in upper limb asymmetry, which is more apparent at slower speeds. To determine if weight distribution or changes in the center of pressure cause muscle activation limb disparities, future research is needed and should include other muscles activated during axillary crutch use such as shoulder flexors and abductors.

While the present findings provide compelling evidence for differences in physical demand and muscle activation while using axillary crutches, there were several limitations. As previously discussed, participants were all healthy, young, and physically active. Most individuals using ADs have some type of physical impairment and/or are aged [3]. Thus, findings in the current investigation may not be fully generalizable to these populations and individuals with acute or chronic physical impairments. However, the use of the postoperative knee immobilizer was chosen in efforts to mimic acute injury for which current data may be most applicable. Only muscles in the lower leg were used for activation measurements along with elbow flexors and extensors. There are a multitude of other muscles important for ambulation with axillary crutches including upper leg and shoulder musculature. It is unknown whether changes in muscle recruitment might be different in other musculature involved in ambulation. Although, previous investigations have shown that functional asymmetry in lower limb joints and musculature below the knee have significant impacts on gait, which in part directed the choice of musculature in the current study [40,41]. Another possible factor which may have influenced upper limb asymmetry is dominance of the upper body. Currently, only the lower limbs were classified for dominance, leaving the contribution of upper limb dominance on the results unknown. This will be important to control for in future investigation as previous findings have shown that upper limb dominance influence limb activity [42]. Lastly, since DOM and NDOM muscle activation was always standardized to BW and not maximum voluntary isometric contraction (MVIC), muscle activation data cannot determine if activation during the DOM and NDOM conditions was different from that of simply walking. However, the central aim to this investigation was to determine whether activation was different based on limb dominance, not mode of ambulation. Attaining valid MVICs also emerged as problematic for some muscles and may have been difficult without performing high numbers of MVIC repetitions for reproducibility [43]. Future research will be needed to determine the generalizability to other populations and muscles and to determine if deviation of muscle activation while using ADs from BW is greater based on limb dominance. In conclusion, physical demands increase while using axillary crutches with limb immobilization regardless of limb dominance. While lower leg muscle activation was similar between dominant and nondominant legs during immobilized crutching, upper limb muscle activation showed asymmetry, which was speed-dependent. However, upper limb asymmetry was independent of limb dominance. This may have important implications in individuals with acute trauma and chronic conditions, whereby interventions may be made to increase symmetry and to possibly influence further injury risk.

## Figures and Tables

**Table 1:** Lower body electromyography (mV) signals standardized to BW (*n* = 12). Data are presented as mean ± SD.

	DOM			NDOM		
Muscle	0.6 mph	0.8 mph	1.0 mph	0.6 mph	0.8 mph	1.0 mph
**Medial Gastrocnemius (MG)**	2.8 ± 0.7	2.7 ± 0.6	2.9 ± 0.9	4.0 ± 2.7	3.5 ± 1.4	3.2 ± 1.2
**Tibialis Anterior (TA)**	2.1 ± 0.8	2.1 ± 0.7	2.3 ± 0.8	2.1 ± 0.8	2.2 ± 0.7	2.2 ± 0.6
**Soleus (SOL)**	3.0 ± 1.4	2.9 ± 1.1	3.1 ± 1.1	3.3 ± 1.1	3.6 ± 1.5	2.9 ± 0.7

**Table 2:** Upper body electromyography (mV) signals standardized to BW (*n* = 12). Data are presented as mean ± SD. * indicates significantly different from 1.0 mph within conditions (*p* < 0.05). # indicates significantly different from 0.6 mph within conditions (*p* < 0.05). † indicates significantly different from CBB at same speed (*p* < 0.05).

	DOM			NDOM		
Muscle	0.6 mph	0.8 mph	1.0 mph	0.6 mph	0.8 mph	1.0 mph
**Ipsilateral Biceps Brachii (IBB)**	6.7 ± 3.5 *†	7.8 ± 3.6 *†	15.0 ± 9.4	7.9 ± 5.8 *†	7.3 ± 4.4 *†	15.8 ± 5.2
**Contralateral Biceps Brachii (CBB)**	14.7 ± 5.6	14.6 ± 6.4	15.1 ± 8.1	15.5 ± 6.8	15.1 ± 5.3	14.2 ± 4.5
**Ipsilateral Triceps Brachii (ITB)**	48.8 ± 20.8	32.8 ± 19.8	34.0 ± 17.9	56.7 ± 38.6	32.1 ± 20.2	35.9 ± 12.3
**Contralateral Triceps Brachii (CTB)**	58.8 ± 34.5	27.5 ± 16.3 #	33.0 ± 15.0 #	50.8 ± 43.4	24.4 ± 12.6 #	24.4 ± 10.0 #

## Data Availability

Data are contained and available within this manuscript.
